# Commentary on: Objective Analysis of Breast Symmetry in Female Patients Undergoing Breast Reconstruction After Total Mastectomy

**DOI:** 10.1093/asjof/ojac091

**Published:** 2022-12-21

**Authors:** Maurice Y Nahabedian

**Affiliations:** Private practice, McLean, VA, USA

See the Original Article here.

Breast symmetry following reconstruction with implants or autologous tissues can be assessed objectively using measurements as well as subjectively using unbiased evaluators. Both are important (Video). In this study, the authors assessed breast symmetry following mastectomy and reconstruction based on 3-dimensional calculation of volume as well as sternal notch to lowest visible point distance.^1^ The lowest visible point was used because not all patients had nipple preservation or reconstruction.

Over the years, I have come to realize that objective, measurement-based breast symmetry is only one component in assessing overall aesthetic outcome of the breast.^2-6^ Equally important is a subjective visual assessment of the breast, based not only on vertical parameters as with this study but also on horizontal parameters. These other parameters include a well-defined sternal cleavage plane, relative positions of the inframammary folds, presence and contour of the lateral mammary fold, lower pole contour, position of the upper border of the breast as well as the slope of the upper pole. Although a patient may have symmetry based on breast volume and lowest visible point distance, they may still have significant asymmetry based on the parameters listed.

The evaluation of breast symmetry based on objective measurements is easy but often does not provide a true representation of actual symmetry. Subjective assessment is more complicated and difficult because there is significant variability based on individuals making the assessments. Assessing symmetry and cosmetic outcome following cosmetic breast augmentation is relatively simple; however, assessing breast symmetry and aesthetic outcome following reconstruction is much more complicated because the natural landmarks are disrupted and recreated. In addition, there can be significant diversity among raters as to what constitutes an ideal outcome. Hypothetically speaking, if a group of unbiased evaluators was assembled to assess breast symmetry and outcome in a given patient, there would be general agreement based on whether the outcome was symmetric and of high quality; however, there would be significant diversity when it comes to analyzing the individual breast parameters. We can look at an outcome and be able to say that symmetry is achieved or not; however, when we micro-analyze the breast, there may be significant differences of opinion based on the different specific parameters. In conclusion, this is a valuable manuscript; however, the readers should be aware that the determination of symmetry and aesthetic outcome is dependent on more than breast volume and the nipple to lowest visible point distance.

## Supplementary Material

ojac091_Supplementary_Data
